# Trends on vibrational spectroscopy tools in the agri-food sector

**DOI:** 10.1007/s00216-025-06166-7

**Published:** 2025-10-18

**Authors:** Candela Melendreras, Jesús Montero, José M. Costa-Fernández, Ana Soldado

**Affiliations:** Department of Physical and Analytical Chemistry, University of Oviedo, Avda. Julián Clavería 8, 33006 Oviedo, Spain

**Keywords:** Non-invasive analysis, Real-time analysis, Near infrared spectroscopy, Raman spectroscopy

## Abstract

Ensuring food safety and quality has become a critical priority in response to increasing global demand and regulatory requirements. To address these challenges, the food industry is demanding sensitive, selective, and robust analytical strategies. Among these, non-destructive spectroscopic sensors (NDSS), particularly those based on vibrational spectroscopy such as near infrared (NIR) and Raman spectroscopy, have demonstrated significant potential for rapid, in situ analysis of food matrices without compromising sample integrity. Current research efforts are focused on the miniaturization and cost-effective design of spectroscopic instrumentation, enabling the development of portable devices suitable for real-time food monitoring across the supply chain. In parallel, advanced chemometric techniques and machine learning algorithms are revolutionizing spectral data interpretation, enhancing model calibration, transferability, and predictive reliability. Artificial intelligence approaches in spectroscopic workflows facilitate the extraction of meaningful patterns from large and complex spectral datasets. Together, these advanced vibrational proposals are converging toward the realization of intelligent, real-time decision-making systems that support sustainable, efficient, and safe food production and distribution, redefining the standards of food quality control in modern agri-food systems.

**Graphical abstract:**

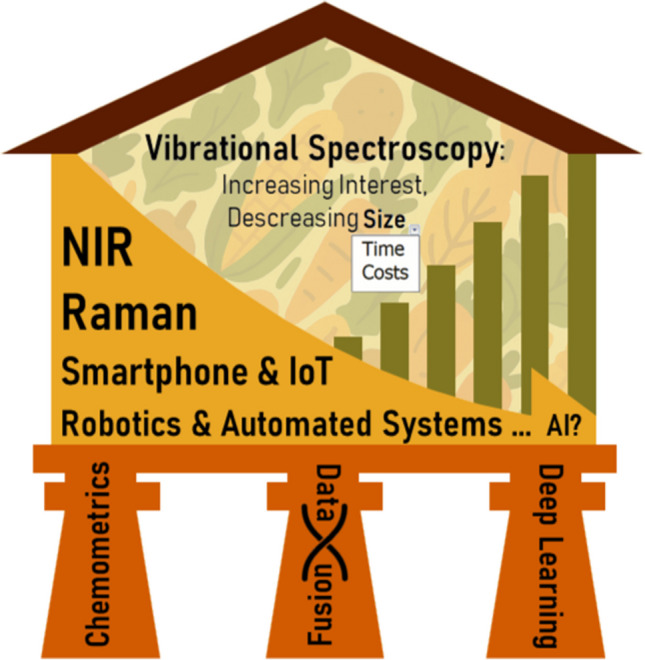

## Introduction

Vibrational spectroscopy has become a key analytical technique in the food industry, providing real-time, non-destructive tools for assessing product quality and safety. Its application supports compliance with regulatory standards and contributes to the broader goals of food safety, consumer protection, and global market integrity. Its relevance in food analysis has grown through integration into non-destructive sensing systems, especially NIRS and Raman spectroscopy. These techniques, supported by chemometrics, enable rapid, multi-parametric assessments and have become essential tools in agri-food applications [1].

**Fig. 1:**
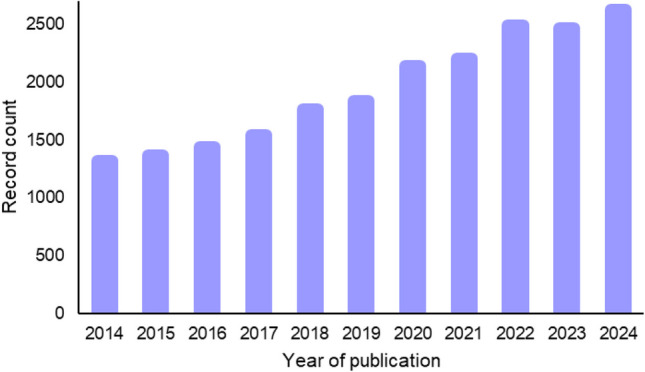
Number of scientific publications (research articles, review papers, and book chapters) from 2014 to 2024 retrieved from Web of Science using the keywords “Near Infrared Spectroscopy” OR “Raman Spectroscopy” combined with “food”

The growing interest in the application of vibrational spectroscopy to food analysis is evident from publication trends over the past decade. A search conducted in Web of Science using the keywords “Near Infrared Spectroscopy” OR “Raman Spectroscopy” combined with “food” yielded over 21,000 scientific publications between 2014 and 2024, including research articles, reviews, and book chapters. This upward trajectory underscores the expanding relevance of these techniques in the agri-food sector, as illustrated in Fig. 1.

The review is structured around two emerging directions in the application of vibrational spectroscopy to food analysis. From an instrumental standpoint, the focus lies on the development and deployment of miniaturized devices [2, 3], which facilitate in situ and on-line measurements. On the data analysis side, the manuscript examines chemometric strategies such as data fusion [4] and deep learning approaches [5]. These methodologies are not mutually exclusive but rather complementary, and their integration is further empowered by artificial intelligence (AI), which extends beyond deep learning to enable automation, real-time decision-making, and smarter spectroscopic workflows.

## Instrumentation: miniaturization and integration

Recent advances in spectroscopic instrumentation have led to the development of compact, portable, and integrated systems tailored for real-time food analysis. These emerging technologies—ranging from miniaturized NIR and Raman devices to handheld and embedded platforms—are reshaping how spectral data is acquired and applied. Their growing relevance reflects a clear need for flexible, field-ready solutions that support rapid, non-destructive, and scalable quality assessment across diverse food environments.

### Miniaturization of NIR devices

The first miniaturized NIR devices featured external radiation sources, required mains power, had a limited wavelength range, low resolution, poor spectral reproducibility, and a small optical window. They also needed to be connected to an external computer, making field applications unfeasible [2]. Technological advances led to the development of medium-sized, handheld sensors incorporating all necessary components for autonomous operation, such as batteries and electronic control systems. The rapid progress in microelectronics and microelectro-mechanical systems (MEMS) has enabled the transition from medium-sized devices (~ 1.5 kg) to microspectrometers (> 100 g) in recent years [6, 7]. The most representative and widely used instruments in miniaturized NIRS are the Phazir™ (Thermo Fisher Scientific) and the MicroNIR (VIAVI). Table 1 summarizes information on some of the most commonly used portable NIR spectrometers. The data were collected from the official websites of the respective manufacturers.

**Table 1:** Main specifications of some commercial handheld NIR spectrometers

Manufacturer	Model	Technology	Spectral resolution (nm)	Spectral range (nm)	Size W × D × H (mm)	Weight (g)
Texas Instruments	NIRscan Nano EVM	Grating-MEMS-DMD	10	900–1700	58 × 62 × 36	100
VIAVI Solutions	MicroNIR 1700	LVF-Linear	12–20	950–1650	50 × 45	116
Si-Ware Systems	NeoSpectra	MEMS-FT	8–16	1250–1700	178 × 91 × 62	1000
Ocean Optics	Flame NIR	Grating	10	970–1700	89.1 × 63.3 × 34.4	265
Hefei SouthNest Technology	NanoFTIR NIR	MEMS Michelson	6	800–2600	143 × 49 × 28	220

*W*, width; *D*, depth; *H*, height; *MEMS*, micro-electromechanical systems; *DMD*, digital micromirror device; *LVF*, linear variable filter; *FT*, Fourier transform

Despite the significant progress in miniaturizing NIR spectrometers, it is important to acknowledge that these devices often exhibit reduced spectral resolution, sensitivity, and signal-to-noise ratio compared to benchtop instruments. Nevertheless, when combined with robust chemometric tools—such as principal component analysis (PCA), partial least squares (PLS), and more advanced modelling techniques—miniaturized systems can still deliver reliable results in agri-food applications focused on quality control and food safety [8]. This synergy has proven effective in detecting food fraud, verifying geographical origin, and identifying adulteration in products like oils, wines, meats, and cereals. To ensure the validity and transferability of these models, it is essential to perform calibration and validation under real-world conditions and to account for the instrumental variability that may exist between different miniaturized devices.

### Miniaturization of Raman devices

Raman spectroscopy is based on the inelastic scattering of monochromatic laser light, which provides information about molecular vibrations through changes in polarizability. It offers a complementary approach to NIR, particularly advantageous for highly hydrated samples, as it minimizes interference from polar O–H bonds and enhances signals from homonuclear groups. Despite its low scattering efficiency—often below 0.0001%—and the resulting challenges in signal-to-noise ratio and acquisition time, recent technological advances have enabled the development of portable and handheld Raman devices [9]. These instruments allow for in-field, real-time analysis of bulk samples without the need for pre-treatment, making them highly suitable for food quality and safety applications. The trend toward miniaturization has led to compact systems that integrate essential optical components and low-power lasers, facilitating their use outside laboratory settings. Table 2 presents information on some of the most widely used portable Raman spectrometers. The technical specifications were obtained from the official websites of the respective manufacturers.

**Table 2:** Main specifications of some commercial handheld Raman spectrometers

Manufacturer	Model	Laser wavelengths (nm)	Spectral resolution (cm^−1^)	Spectral range (cm⁻^1^)	Size W × D × H (mm)	Weight (kg)
Agilent	Resolve	830	15	350–2000	155 × 73 × 290	2.2
Metrohm	MIRA XTR	785	8–10	400–2300	88.2 × 45.3 × 125.5	0.7
B&W Tek (Metrohm)	TacticID-1064 ST	1064	11	176–2500	212 × 107 × 55	1.55
Rigaku	Progeny	1064	8–10	200–2500	74 × 81 × 299	1.5
Thermo Fisher	TruScan	785	8–10.5	250–2875	118 × 46 × 199	0.95

*W*, width; *D*, depth; *H*, height

To address the intrinsic limitations of conventional Raman spectroscopy—particularly its low scattering cross-section and sensitivity—several advanced techniques have been developed. One of the most prominent is surface-enhanced Raman scattering (SERS), which amplifies signal intensity by several orders of magnitude through the use of metallic nanostructures placed in close proximity to the analyte. This enables the detection of trace-level contaminants such as pesticides and adulterants. As detailed in the review by Zhou et al. [10], the article not only explains the theoretical foundations of SERS but also presents numerous application examples for the detection of heavy metals, foodborne pathogens, illegal additives, biotoxins, pesticides, and veterinary drug residues in food products. The potential of miniaturized Raman systems and SERS-based techniques is significantly enhanced when combined with decentralized analytical strategies such as microfluidics. These integrated platforms enable rapid, sensitive, and on-site detection of contaminants in complex food matrices. Microfluidic-SERS systems have demonstrated great promise for the detection of a wide range of food contaminants [11].

### Portable applications: smartphone integration and IoT connectivity

In recent years, there has been a growing shift in the application of spectroscopic techniques, such as NIR and Raman, toward more portable and decentralized formats. One of the most active areas of development is the miniaturization of instruments, with particular emphasis on the integration of micro-spectrometers into smartphones and other mobile platforms [12]. This trend reflects the increasing demand for analytical tools that can operate outside traditional laboratory settings, enabling on-site, real-time decision-making in fields such as food safety and agriculture.

Alongside miniaturization, another key direction is the digital integration of these techniques through cloud computing and Internet of Things (IoT) technologies. These infrastructures make it possible to store, manage, and process spectral data remotely, even when collected from multiple points along a production line or from geographically dispersed locations [13]. However, despite the rapid development of advanced chemometric methods, many instruments still lack software capable of supporting these innovations.

### Industrial-scale integration: robotics and automated spectroscopic systems

Another key direction in the miniaturization of spectroscopic techniques is their integration into automated production environments. Instead of focusing only on handheld or field-deployable devices, current research is increasingly exploring how compact NIR and Raman sensors can be embedded into robotic systems, conveyor lines, and smart manufacturing platforms. This approach enables continuous, real-time quality control without interrupting the production flow [6].

The combination of optical sensors, robotics, and machine learning algorithms allows for advanced food quality assessment with high precision and minimal human intervention. These integrated systems can detect subtle variations in product composition, identify contaminants, and even make autonomous decisions based on spectral data [14]. However, ensuring metrological traceability in such process-integrated technologies is critical to guarantee that the measurements are reliable, comparable, and suitable for regulatory and safety decisions, especially when deployed outside traditional laboratory settings [15].

## Chemometrics: data fusion and deep learning

In chemometric analysis, it is useful to separate data fusion and deep learning approaches, as they rely on different methodologies. Data fusion integrates information from multiple spectral sources using classical multivariate techniques, while deep learning uses neural networks to model complex patterns in spectral data. Each offers distinct advantages in food analysis and merits individual discussion.

### Data fusion strategies

Data fusion has emerged as a pivotal strategy in chemometric analysis, particularly in the context of vibrational spectroscopy applied to agri-food systems. As miniaturized and portable NIR and Raman instruments become increasingly prevalent, the need to enhance the analytical performance of these devices has driven the development of robust multivariate data integration techniques. Chemometric data fusion enables the combination of complementary information from multiple sensors or modalities, improving model accuracy, reliability, and generalizability [4, 16].

**Fig. 2:**
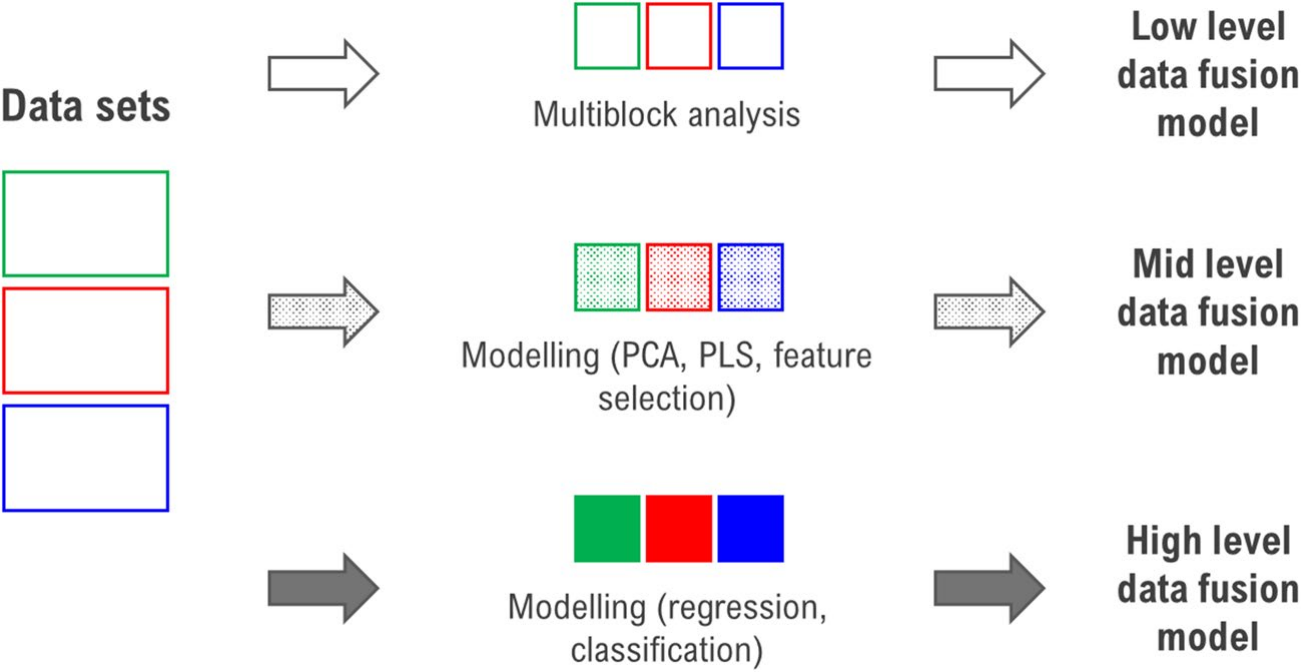
Graphical summary of three types of data fusion

Data fusion can be categorized into three levels (Fig. 2): low-level fusion, which merges raw data matrices; mid-level fusion, which integrates extracted features such as principal components or selected variables; and high-level fusion, which combines outputs from individual models. Each level offers distinct advantages depending on the complexity of the dataset and the analytical objective.

In food analysis, mid-level fusion is particularly effective, as it allows for dimensionality reduction and noise filtering prior to integration, enhancing the interpretability and stability of predictive models [17].

In the literature, several studies have demonstrated the robustness of mid- and high-level data fusion strategies in food analysis when integrating spectral data from diverse non-destructive sensors. For example, Zhang et al. [18] combined NIR, fluorescence, and laser-induced breakdown spectroscopy (LIBS) data to classify the origin of edible gelatin, achieving high accuracy through feature-level fusion. Li et al. [19] applied several high-level data fusion strategies to improve the quantitative detection of honey adulteration, integrating the outputs of multiple spectroscopic models to enhance prediction accuracy and model stability. In the dairy industry, a review [20] highlights the use of data fusion to integrate NIR and mid-infrared (MIR) spectroscopy, improving the prediction of milk composition and quality parameters. These applications confirm the value of data fusion in building robust chemometric models for real-time, non-destructive food analysis.

The integration of data fusion into miniaturized spectroscopic systems is pivotal for on-site and in-line applications requiring fast, non-destructive analysis. By combining complementary data sources, portable instruments can reach analytical performance comparable to benchtop setups, enabling real-time decision-making in quality assurance and food safety. Its implementation enhances the analytical robustness of compact devices and facilitates their deployment in operational environments, driving the digitalization of the agri-food sector.

### Deep learning strategies

**Fig. 3:**
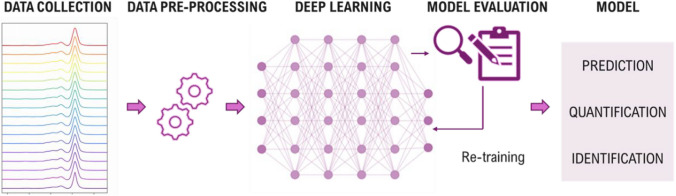
Simplified deep learning workflow for spectroscopic analysis

Deep learning is a branch of machine learning that builds upon multilayer artificial neural networks (ANNs), extending their architecture to deeper, more complex models capable of learning hierarchical representations from data. These models consist of interconnected layers of artificial neurons that transform input data through weighted connections and non-linear activation functions. During training, the network adjusts these weights to minimize prediction error using algorithms such as back-propagation and gradient descent. This hierarchical structure enables the automatic extraction of relevant features from raw data, eliminating the need for manual preprocessing. Deep learning offers robust capabilities for analyzing complex, high-dimensional spectral data. In spectroscopic applications such as NIR and Raman, these models effectively capture non-linear relationships and subtle spectral features, often outperforming traditional chemometric approaches. Their ability to enhance classification, prediction, and anomaly detection makes them particularly valuable in food analysis, where rapid and reliable assessment of quality and authenticity is critical. To maintain model accuracy and adaptability under changing biological and environmental conditions, periodic evaluation and retraining with updated spectral datasets are essential. This ensures sustained performance, mitigates the effects of instrumental drift and sample variability, and supports reliable deployment in real-world agri-food systems. Figure 3 outlines the deep learning workflow in spectroscopic models.

While traditional ANNs have been successfully applied to spectral data analysis—particularly for classification and regression tasks—deep learning models such as convolutional neural networks (CNNs) and recurrent neural networks (RNNs) offer enhanced performance in handling high-dimensional, non-linear spectral features. These architectures enable automatic feature extraction and improved generalization, making them especially suitable for complex agri-food applications involving biological variability and heterogeneous sample matrices.

ANNs have been widely applied in food analysis using vibrational spectroscopy. These models have demonstrated effective performance in tasks like classification and quantification, particularly in detecting compositional differences and assessing food quality [21]. For instance, ANN-based approaches have been used to quantify adulterants in powdered dairy products using Raman imaging [22]. Similarly, NIR spectroscopy combined with non-linear algorithms such as back-propagation ANN has demonstrated high accuracy in estimating quality parameters of frozen food, including drip loss and texture, outperforming linear models [23].

Building upon the successful use of ANNs in food analysis, deep learning architectures have further expanded the capabilities of vibrational spectroscopy by enabling more robust and automated interpretation of complex spectral data. These models have demonstrated superior performance. Deep learning has been widely applied to food classification and identification tasks using vibrational and optical spectroscopy. For instance, Raman spectroscopy combined with deep learning architectures enables the automated recognition of molecular patterns to distinguish between oil types and detect adulteration, which is particularly valuable in quality control environments requiring rapid screening [24]. Similarly, by integrating data from ultraviolet-visible-near-NIR reflection spectroscopy and fluorescence spectroscopy, deep learning models can classify food items based on attributes such as freshness, contamination, or physical defects, supporting reliable and high-throughput inspection systems [25].

Deep learning has shown significant promise for non-destructive quantification of food quality attributes using NIRS and hyperspectral imaging, thanks to its ability to model complex spectral patterns and extract relevant features without the need for manual preprocessing [26]. This approach enables more accurate and efficient estimation of key parameters in diverse food matrices, supporting rapid and reliable quality assessment.

Despite its strong performance in spectral modelling, deep learning presents notable limitations, particularly regarding model interpretability. Unlike conventional chemometric approaches that offer transparent insights into variable relevance, deep learning models often operate as black boxes, hindering the traceability and justification of predictions. This lack of transparency poses challenges in regulated domains such as food analysis, where accountability is essential. Therefore, its application should be supported by rigorous validation, benchmarking against established methods, and a critical assessment of its added value [5].

## Artificial intelligence in spectroscopic workflows

The integration of artificial intelligence (AI) into spectroscopic workflows is evolving beyond algorithmic performance, demanding a broader perspective that includes usability, transparency, and trust. While deep learning has demonstrated strong predictive capabilities, its adoption in food analysis remains constrained by limited data availability, lack of standardized validation protocols, and concerns over reproducibility. Moreover, the opacity of many AI models challenges their acceptance in regulated environments, where interpretability and traceability are essential.

To ensure meaningful deployment, AI systems must be designed with end-users in mind—offering intuitive interfaces, actionable outputs, and clear documentation of model behavior. This user-centered approach is particularly relevant in the food industry, where stakeholders range from laboratory technicians to regulatory authorities. Additionally, the ethical and legal dimensions of AI—such as data privacy, algorithmic bias, and accountability—must be addressed through collaborative frameworks that involve both developers and domain experts [27].

### State of the art and critical assessment

Vibrational spectroscopy technologies are increasingly being integrated into smart sensor systems, contributing to the digital transformation of agriculture. Smart sensor technologies are shaping the future of precision agriculture by enabling data-driven decision-making, resource optimization, and sustainability [28]. Non-destructive approaches like NIRS and Raman are particularly valuable for assessing fruit and vegetable maturity, offering rapid and reliable alternatives to traditional destructive methods [29]. Strategies such as experimental design, variable selection, spectral preprocessing, and biomarker identification have proven essential for both regression and classification tasks. The integration of spectroscopic techniques with machine learning—and more recently, deep learning—has significantly enhanced analytical performance by enabling the extraction of meaningful patterns from complex data. This synergy supports the development of scalable, automated decision-support systems, positioning chemometrics as a cornerstone of Agriculture 4.0 [30].

Information fusion has emerged as a key advancement in vibrational spectroscopy, addressing the limitations of single-method analysis. By integrating data from complementary techniques—such as infrared, fluorescence, and Raman spectroscopy—multimodal approaches enhance the precision, reliability, and scope of non-destructive assessments. This strategy enables more comprehensive sample characterization and supports the development of robust analytical systems for agri-food applications.

However, several limitations remain. Biological variability affects model robustness, and the absence of standardized calibration protocols, along with the need for large, high-quality datasets, limits model transferability [31]. Additionally, the black-box nature of deep learning raises concerns in regulated environments where interpretability and traceability are critical. While hardware costs have decreased, implementation still requires technical expertise and investment, posing challenges for small-scale producers. Ultimately, the value of AI in spectroscopy lies not only in computational performance but in its ability to enable transparent and responsible decision-making across the food value chain [27].

### Outlook

Looking forward, the future of NIRS and Raman spectroscopy in the agri-food sector is closely linked to the development of intelligent, connected systems. Integration with IoT platforms, cloud computing, and edge analytics will enable continuous, autonomous monitoring of food quality and safety. Czaja and Engelsen [32] argue that NIRS, in particular, represents a green analytical technology well-suited for circular economy models due to its low environmental impact and versatility. Furthermore, democratizing access to these technologies—through open-source tools, affordable hardware, and user-friendly interfaces—will be essential to ensure widespread adoption. Interdisciplinary collaboration among engineers, data scientists, and food technologists will be key to overcoming current limitations and advancing toward a more sustainable, transparent, and efficient agri-food system.

## Data Availability

Data will be made available on request.

## Abbreviations

ANNs: Artificial neural networks
IoT: Internet of Things
LIBS: Laser-induced breakdown spectroscopy
MEMS: Microelectron-mechanical systems
MIR: Medium infrared
NDSS: Non-destructive spectral sensors
NIR: Near infrared
NIRS: Near infrared spectroscopy
PCA: Principal component analysis
PLS: Partial least squares regression
SERS: Surface-enhanced Raman scattering
